# Success in the Medical Profession: An Introductory Lecture, Delivered at the Mass. Med. College

**Published:** 1851-04

**Authors:** John Ware

**Affiliations:** Hersey Professor of the Theory and Practice of Medicine in Harvard University.


*Success in the Medical Profession: An Introductory Lecture, delivered at the Massachusetts Medical College, Nov. 6, 1850.* By JOHN WARE, M. D., Hersey Professor of the Theory and Practice of Medicine in Harvard University.

THIS is admirable. If it did not savor of presumption, we would say Dr. Ware is a man after our own heart. His views are our views: they have always been our views; but we were beginning to fear that we were alone in our thinking. Suddenly and unexpectedly we open an unpretending pamphlet, and there stand out our own thoughts, gracefully adorned, strong, robust, majestic. There they are—what we have often thought, but ne'er so well expressed; and right glad we are to see them.

*"It is too often overlooked that the final purpose of all medical study is practice."* Verily, it is. *"A man may know a vast deal of the profession, and yet be a very poor practitioner."* Yes, indeed, he may know a great deal of what is supposed to be science, and somewhat too of real knowledge, and very little about sick people—and as long as the microscope receives more attention than the lancet, the evil will inerease upon us.

There is "a *distinction between a pathological* and a *therapeutical diagnosis*"—a *distinction!* yes, a distinction so wide as to create an entire difference. The one is mainly a matter of curiosity; the other, a matter of life and death. The one can be taught by professors who never had practice, and never knew how to practice; the other, by men who can cure disease. But the evil day is upon us. Woe to us that we dwell in Marcheck! Cry out, Dr. Ware, and spare not! There is need of men of common sense and uncommon nerve about this time.

